# Alterations in intestinal microbiota and enzyme activities under cold-humid stress: implications for diarrhea in cold-dampness trapped spleen syndrome

**DOI:** 10.3389/fmicb.2023.1288430

**Published:** 2023-11-10

**Authors:** Yi Wu, Na Deng, Jing Liu, Ping Jiang, Zhoujin Tan

**Affiliations:** ^1^College of Traditional Chinese Medicine, Hunan University of Chinese Medicine, Changsha, China; ^2^The First Affiliated Hospital of Hunan University of Chinese Medicine, Changsha, China

**Keywords:** intestinal microbiota, digestive enzyme activities, diarrhea, cold-dampness trapped spleen syndrome, Chinese medicine

## Abstract

**Introduction:**

Cold and humid environments alter the intestinal microbiota, and the role of the intestinal microbiota in the development of diarrhea associated with cold-dampness trapped spleen syndrome in Chinese medicine is unclear.

**Methods:**

The 30 mice were randomly divided into normal and model groups, with the model group being exposed to cold and humid environmental stresses for 7 days. Then, mouse intestinal contents were collected and analyzed their intestinal microbiota and digestive enzymes.

**Results:**

Our findings revealed significant increases in sucrase and lactase activities, as well as microbial activity, in the model group (*p* < 0.05). β-diversity analysis highlighted distinct intestinal microbiota compositions between the two groups. Specifically, the experimental group showed a unique dominance of the genera and strains *Clostridium sensu stricto* 1 and *Clostridium* sp. ND2. LEfSe analysis identified *Helicobacter*, *Roseburia*, and *Eubacterium plexicaudatum* ASF492 as differentially abundant species in them model group. Network analysis demonstrated that rare bacterial species mostly governed the microbial interactions, exhibiting increased mutual promotion. On the other hand, abundant species like *Lactobacillus johnsonii* and *Lactobacillus reuteri* showed mutual inhibitory relationships.

**Discussion:**

In summary, exposure to cold and humid conditions led to increased intestinal enzyme activities and a shift in microbial composition, favoring the growth of rare bacterial species. These changes suggest that rare bacteria in the intestinal microbiota play a critical role in the pathology of diarrhea associated with cold-dampness trapped spleen syndrome, revealing unique survival strategies among bacterial populations under stressful conditions.

## 1. Introduction

Cold or humid environments are considered extreme conditions in both nature and human society, and they have been shown to adversely affect intestinal function and induce physiological and immune responses in animals (Scharf et al., 2019; Huang B. et al., 2022). There is a growing body of evidence indicating that cold or rainy conditions can amplify the incidence of diarrhea (Xu et al., 2013; Thongprachum et al., 2015; Thiam et al., 2017; Huang et al., 2021; Zolotova et al., 2021). This phenomenon is particularly observed in livestock, where it is referred to as “cold stress diarrhea” (Gou et al., 2021; Sun et al., 2022; Zhou et al., 2022). Research supports the premise that intestinal microbes significantly contribute to maintaining intestinal stability under such trying conditions. For instance, in harsh climates like highlands or polar regions, microbial species and survival strategies evolve to adapt to cold stress or combined cold and wet stress (Huang X. et al., 2022; Marian et al., 2022). It’s also been found that prenatal exposure to cold stress can alter the intestinal microbiota composition in mice (Zheng et al., 2020). Moreover, cold stress conditions have been shown to reduce the abundance of intestinal microorganisms in *Pelteobagrus fulvidraco*, simultaneously spurring a rapid increase in pathogenic bacteria, highlighting potential harm to intestinal health instigated by the cold environment (Hu et al., 2022).

In Traditional Chinese Medicine (TCM), “humidity” is intricately linked with diarrhea. Chronic or intermittent exposure to cold and humid weather is regarded as detrimental to human health and is referred to as a “seasonal pathogen” in China. Another critical factor associated with diarrhea is the failure of the spleen’s transportation function. Within the TCM paradigm, the “spleen” encompasses functionalities that, in Western medicine, might equate to components or the entirety of the digestive and immune systems. The “transportation and transformation” function of “spleen” in Chinese medicine means the function of the spleen by which the essence is transformed from food and drink, absorbed, and distributed to all parts of the body. Our prior studies have effectively devised a mouse model for diarrhea associated with the dampness-heat syndrome. This model was achieved through the combined stresses of a hot and humid environment and the administration of lard gavage. Our findings, as reported in Li et al. (2021), emphasize the significant role of intestinal microorganisms in the diarrhea associated with dampness-heat syndrome in mice. The study found that diarrhea reduced the abundance of *Lactobacillus gasseri*, *Enterobacterium lactis*, *Lactobacillus roxellanae*, *Lactobacillus vaginalis*, and *Curvibacter lanceolatus* in the intestine, increased the abundance of *Muribaculum intestinale*, *Lactobacillus curvatus*, *Lactobacillus gastricus*, and *Staphylococcus epeidermidis*, *Lactobacillus gasseri* was is considered to be the key species for the identification of diarrhea with the intestinal dampness-heat syndrome. Diarrhea triggered by cold and humid environmental conditions is termed “diarrhea with cold-dampness trapped spleen syndrome” in TCM. However, the micro-ecological shifts accompanying this syndrome remain largely uncharted.

Therefore, the primary objective of this study was to investigate the impact of cold and humid environments on the transformation of intestinal bacteria. Specifically, we aimed to gain valuable insights into the biological processes underlying diarrhea with the cold-dampness trapped spleen syndrome in Chinese medicine. Furthermore, our findings have the potential to contribute to the development of more accurate diagnostic techniques and effective therapeutic approaches in the field.

## 2. Materials and methods

### 2.1. Experimental animals and husbandry environment

Four-week-old SPF-grade male KM mice (Wu et al., 2022) (*n* = 30, 18–20 g) sourced from Slack Jingda Experimental Animal Co, Ltd. (Changsha, China), were housed at Hunan University of Chinese Medicine under controlled conditions (23–25°C, 47–53% humidity), with *ad libitum* access to food and water. Animal production license number: SCXK (Xiang) 2019–0004. Facility use license number: SYXK (Xiang) 2019–0009.

### 2.2. Experimental materials and reagents

Artificial climate chamber from Tianling Instruments Co., Ltd. (Jiangsu, China), vertical pressure steam sterilizer from Yamato Technology Co., Ltd. (Chongqing, China), type 722 visible spectrophotometer from Shunyu Hengping Scientific Instruments Co., Ltd. (Shanghai, China), desktop high-speed frozen centrifuge from Pingfan Instrument Co., Ltd. (Changsha, China), constant temperature shaker incubator from Jintan Ronghua Instrument Manufacturing Co., Ltd. (Changzhou, China).

Acetone from Hunan Huihong Reagent Co., Ltd. (Changsha, China), fluorescein diacetate (FDA) and 2-nitrophenyl-β-D-galactopyranoside (ONPG) from Yuanye Biotechnology Co., Ltd. (Shanghai, China), 3,5-dinitrosalicylic acid from Runcheng Biotechnology Co., Ltd. (Shanghai, China).

### 2.3. Modeling

After 3 days of acclimatization feeding, the mice were randomly divided into the normal group (CW-Cc, *n* = 15) and the model group (CW-Mc, *n* = 15). The CW-Mc group was exposed to an artificial climate chamber for seven consecutive days from 8:00 to 12:00, maintaining a temperature of (4 ± 0.5)°C and humidity of (90 ± 2)%. The CW-Cc group was raised in an environment with a temperature of (24 ± 1)°C and humidity of (50 ± 3)%. Throughout this period, both groups had unrestricted access to water, but they were not provided with food. Successful model establishment was confirmed by observing increased defecation frequency, moist feces, disheveled hair, and signs of mental fatigue in the CW-Mc group mice.

### 2.4. Sample collection

After modeling success, we selected 10 mice with serious diarrhea of similar severity in the CW-Mc group, five of which were studied for microbial sequencing and the rest for intestinal enzyme activity. All mice were sacrificed by rapid neck dissection, and the section from the duodenum to the terminal ileum was dissected on an ultra-clean operating table, the contents of the intestinal section were collected under sterile conditions following the reference (Wu et al., 2021). Samples in the CW-Cc group were marked as CW-Cc 1 through CW-Cc 5, samples in the CW-Mc group were marked as CW-Mc 1 through CW-Mc 5, these samples were promptly stored in a −80°C refrigerator for subsequent microbial sequencing.

### 2.5. Intestinal enzyme activity and microbial activity detection

Enzyme activity measurements were conducted based on the methods established by Li et al. (2023). Intestinal content samples from five mice in each group were mixed, and then transferred to separate centrifuge tubes containing glass beads and an appropriate volume of sterile water. The mixture was then vigorously shaken on a vortex mixer for 10 min to ensure thorough enzyme release. Subsequently, it was centrifuged at 3,000 rpm for 8 min. The resulting supernatant was collected as the crude enzyme solution for testing. Each sample was subjected to three repetitions. Amylase and sucrase activities were determined using the DNS colorimetric method, defining one enzyme activity unit (U) as the generation of 1 mg of reducing sugar from 1 g of intestinal contents/mucosa at 40°C for 15 min. Lactase activity was determined using the ONPG method, defining one enzyme activity unit (U) as the decomposition of 20 mmol/L substrate by 1 g of intestinal contents/mucosa at 37°C for 10 min. Microbial activity was assessed using the fluorescein diacetate (FDA) method (Schnürer and Rosswall, 1982; Tang et al., 2020), defining one enzyme activity unit (U) as the breakdown of 2 mL of substrate at a concentration of 10 μg/mL per 1 g of intestinal contents/mucosa at 24°C for 90 min.

### 2.6. DNA extraction, sequencing, and data pre-processing

DNA was extracted from the collected samples using the Meiji kit (HiPure Soil DNA Mini Kit, D3142-03). The quantity and quality of the DNA were assessed using NanoDrop ND-2000 spectrophotometer and Qubit 3.0 fluorescence quantitation instrument, as well as agarose gel electrophoresis. PCR amplification of the bacterial 16S rRNA gene was performed using specific primers (27F and 1492R), and the PCR products were purified and quantified. The purified samples were then mixed according to the desired sequencing volume. Libraries were generated using the SMRTbell Template Prep Kit 1.0-SPv3 and sequenced on the PacBio platform using DNA Polymerase Binding Kit 2.0. Raw sequencing data were preprocessed using quality filtering, paired-end sequence assembly, and chimera removal, primarily using Qiime (Caporaso et al., 2010) and mothur software.

### 2.7. Bioinformatics

Operational Taxonomic Unit (OTU) clustering was performed using USEARCH software (Bokulich et al., 2013; Edgar, 2013) at a 97% similarity threshold, followed by comparison and annotation of OTUs using the greengene database by blast method. The analysis included the generation of Venn diagrams using R language and measurement of α diversity indices (Chao 1, ACE, Shannon and Simpson) using MOTHUR software and R language (Shannon, 1948; Simpson, 1949; Chao, 1984). PCoA analysis and visualization using R software. Species distribution at different taxonomic levels (phylum, genus, species) based on OTU data was visualized using species distribution histograms and sankey diagrams. Relative abundance histograms were drawn using Graph pad Prism 9.0, and sankey diagram was drawn using the R software ggalluvial package (Rosvall and Bergstrom, 2010).

Linear discriminant analysis Effect Size (LEfSe) analysis, which combines statistical analysis and data dimensionality reduction, was applied to identify significant biomarkers. LEfSe analysis included evolutionary branch diagrams, LDA value distribution histograms, and abundance diagrams of biomarkers in different sample groups (Segata et al., 2011). Random forest variable importance analysis was performed using R language software. Spearman correlation analysis was conducted to calculate the species correlation coefficient. Gephi 1.0.3.8 software was used to visualize network co-occurrence based on the correlation analysis. Bacterial function prediction analysis was carried out by using PICRUSt 2 software and KEGG and METACY databases.

### 2.8. Statistical analysis

The continuous variables data were expressed as mean ± standard deviation. Statistical data were analyzed using SPSS 24.0 software (IBM, Almonk, NY, United States). Independent sample *t-*test was used when the data followed normal distribution and homogeneity of variance. Nonparametric tests, specifically the Mann–Whitney U test, were employed when the data did not meet the assumptions of normality and homogeneity of variance. A significance level of *p* < 0.05 or *p* < 0.01 was used as the threshold for statistical significance.

## 3. Results

### 3.1. Intestinal digestive enzyme activity and microbial activity of diarrhea mice

**Figure 1:**
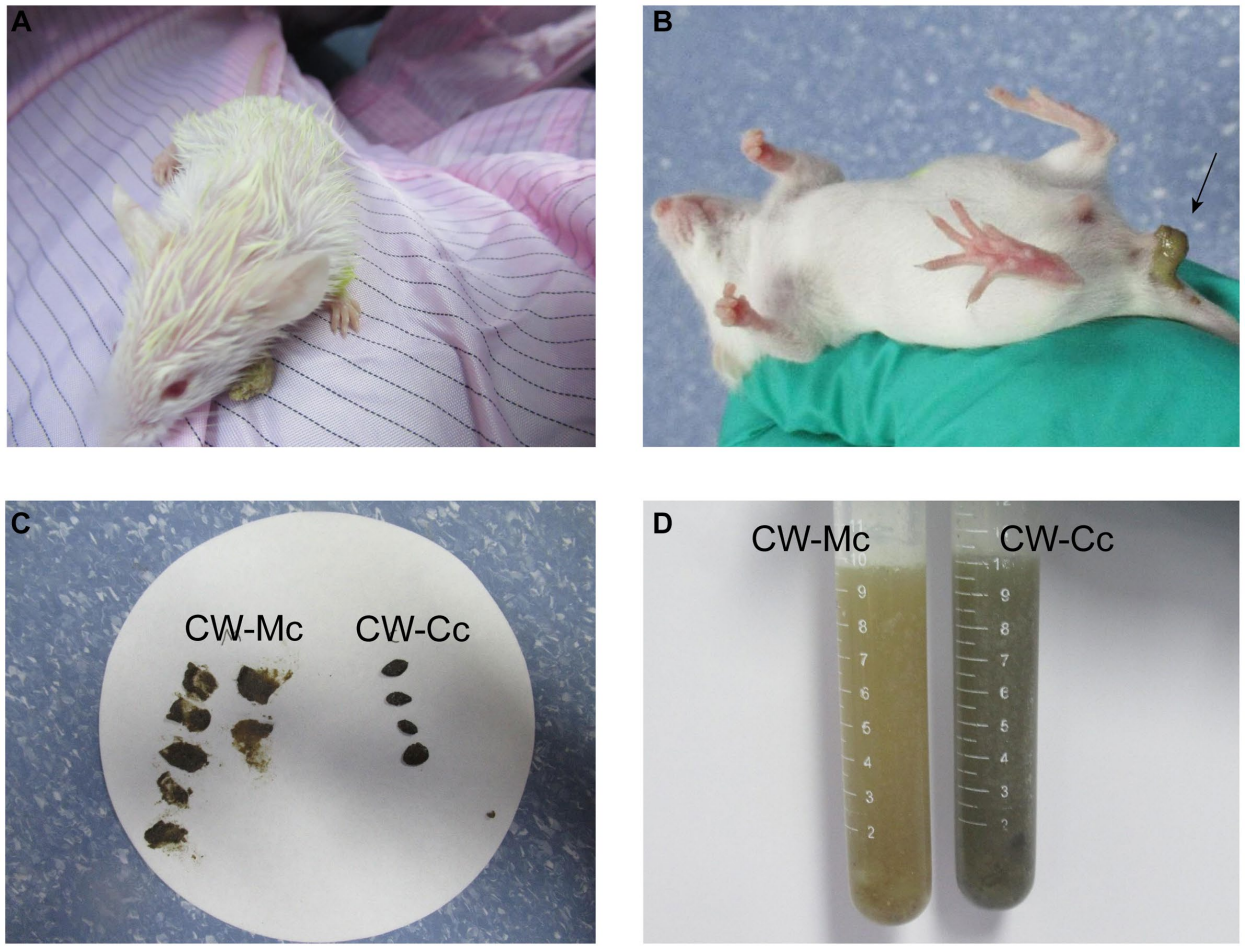
Mouse general status chart. **(A)** The mental status of mice after cold and humid environmental stress. **(B)** Fecal characteristics of mice in the CW-Mc Group. **(C)** Comparison of fecal traits and texture between two groups of mice. **(D)** The color of the fecal water solution between the two groups.

**Figure 2:**
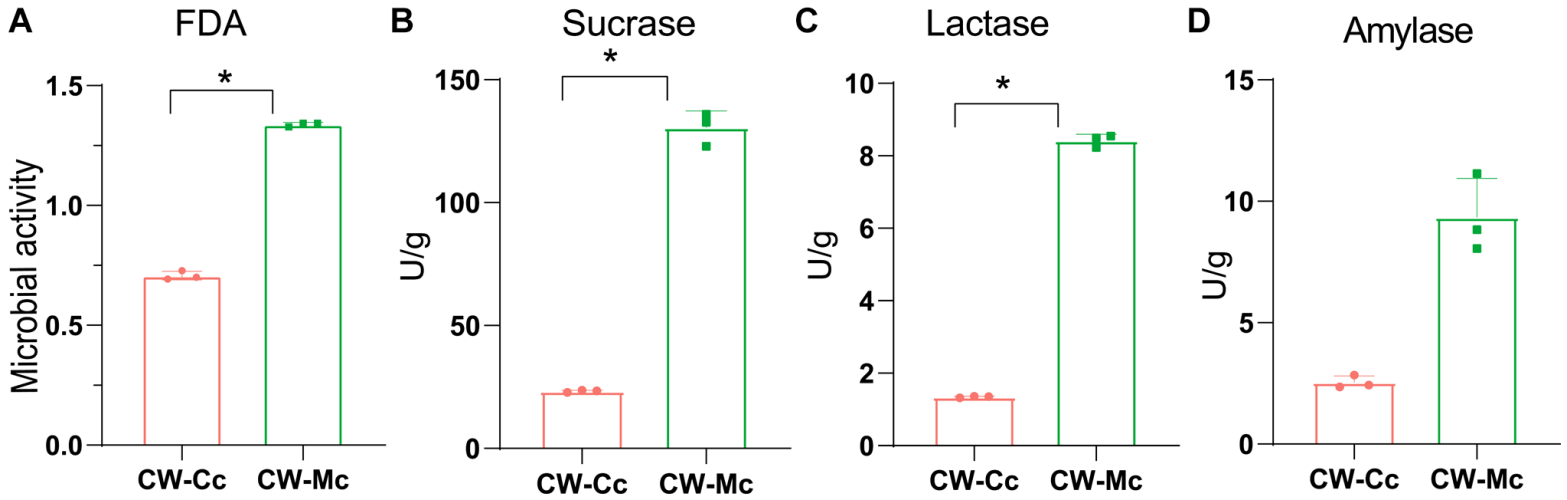
Effects of diarrhea induced by cold and humid environmental stresses on the intestinal content enzyme activities and microbial activity. **(A)** Microbial activity. **(B)** Sucrase activity. **(C)** Lactase activity. **(D)** Amylase activity. Data were expressed as mean ± standard deviation, *n* = 5, *p < 0.05.

**Figure 3:**
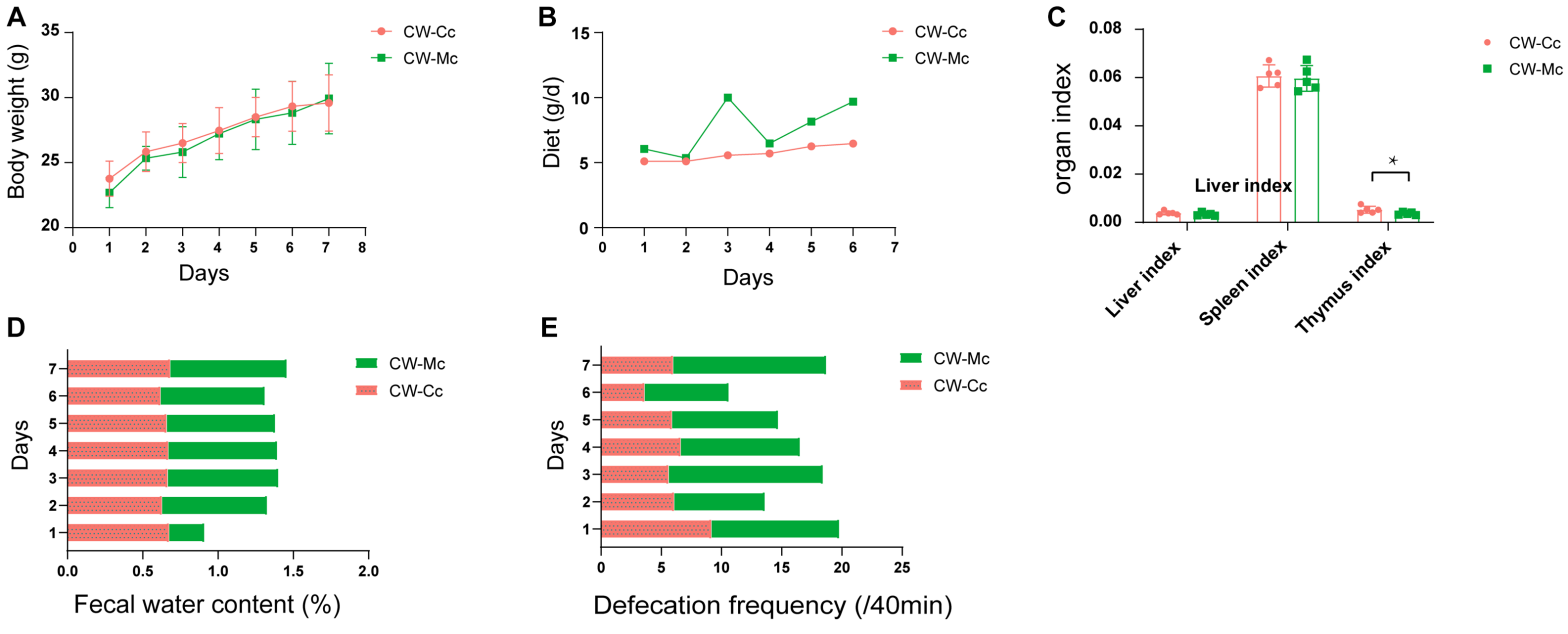
**(A)** Body weight (g). **(B)** Daily food intake. **(C)** Liver index, spleen index and thymus index. **(D)** Fecal water content (%). **(E)** Defecation frequency (/40 min) of mice during the modeling period. Data of body weight and organ index were expressed as mean ± standard deviation, *n* = 5, *p < 0.05.

As shown in Figure 1, the mice in the CW-Mc group had poor spirit and disheveled hair after cold and humid environment stress, had unformed excrements with high water content that were sticky when pressed. After the model has been established, the color of the fecal water in the CW-Mc group was significantly lighter than that in the CW-Cc group under the same concentration of fecal water solution. In Figure 2, the sucrase activity, lactase activity and microbial activity of the intestinal contents of the CW-Mc group were significantly higher compared to the CW-Cc group (*p* < 0.05). As shown in Figures 3A–C, compared with the CW-Cc group, there was no significant difference in body weight of the CW-Mc group (*p* > 0.05), food intake was considerably greater in the CW-Mc group, and the spleen index, the thymus index were lower in the CW-Mc group (*p* > 0.05, *p* < 0.05). The fecal water content and defecation frequency (/40 min) in mice can objectively suggest mice diarrhea. Figures 3D,E displayed that during the modeling period, compared with the CW-Cc group, the fecal water content in the CW-Mc group was slightly higher from the second day, and the defecation frequency in the CW-Mc group was significantly higher.

### 3.2. The model affects the number of intestinal contents microbiota OTUs

**Figure 4:**
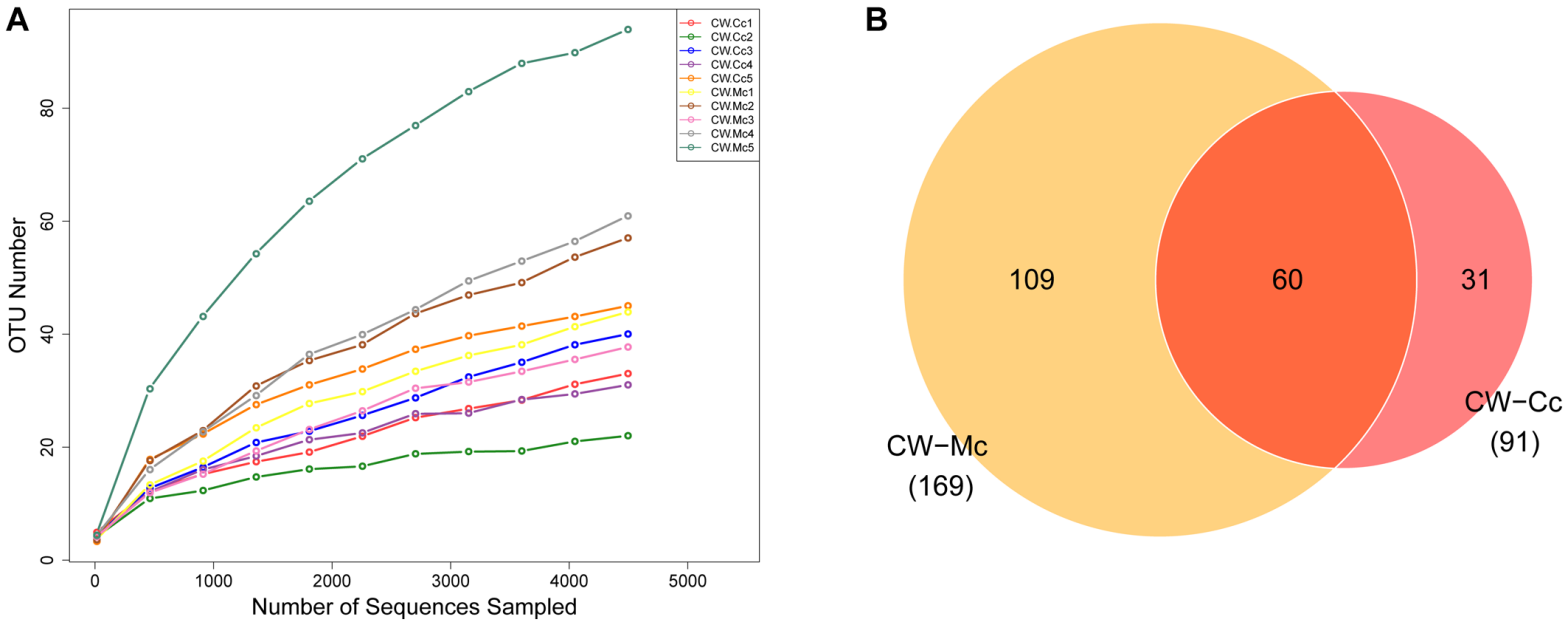
**(A)** Species dilution curves: the horizontal coordinate indicates the amount of randomly selected sequencing data and the vertical coordinate indicates the number of OTUs observed. **(B)** Venn diagram: based on the number of OTUs.

As the number of sequencing increases, the growth rate of the number of OTUs decreases, which means the amount of sequencing data can meet this analysis (Figure 4A). As shown in Figure 4B, a total of 90,064 high-quality bacterial 16S rRNA sequences were obtained by sequencing, including 45,990 in the CW-Cc group and 44,075 in the CW-Mc group. A total of 260 OTUs were clustered and analyzed from the 16S rRNA sequences of all samples, including 91 in the CW-Cc group with 31 unique OTUs and 169 in the CW-Mc group with 109 unique OTUs, for a total of 60 OTUs in the two samples.

### 3.3. α and β diversity of intestinal contents microbiota involved in diarrhea caused by cold and humid environmental stresses

**Figure 5:**
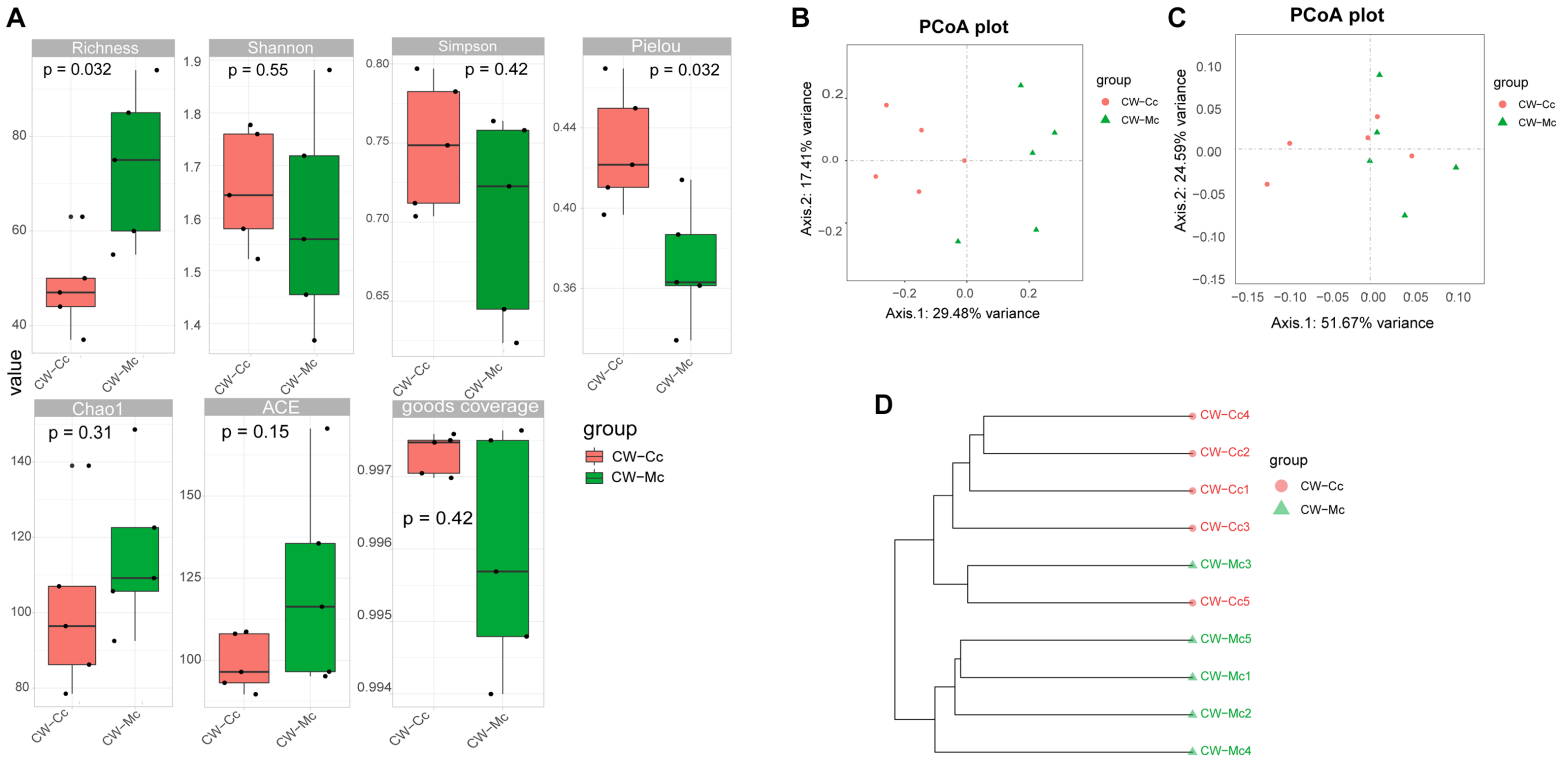
**(A)** α Diversity index calculation based on OTU classification levels by Richness, Shannon, Simpson, Pielou, Chao 1, ACE, and goods coverage, respectively. **(B)** The PCoA analysis based on unweighted Unifrac distance. **(C)** The PCoA analysis based on weighted Unifrac distance (where the dots indicate each sample separately; different colors represent different groupings; the closer the distance of the dots, the more similar the compositional structure between each group). **(D)** Tree structure constructed using the Unweighted pair group (averaging method UPGMA algorithm based on bray curtis distance).

Compared with the CW-Cc group, the Richness index was significantly higher (*p* < 0.05), and the ACE index Chao1 index was higher (*p* > 0.05) in the CW-Mc group. Compared with the CW-Cc group, the Pielou index in the CW-Mc group was significantly lower (*p* < 0.05), the Simpson index and Shannon index were also lower (*p* > 0.05) (Figure 5A). β diversity analysis based on the unweighted Unifrac distance only consider the presence or absence of microbial species in the community, while the weighted Unifrac distance consider both the presence or absence of microbial species and their abundance in the respective communities. We first performed an unweighted group-averaged cluster analysis (Figure 5D), which visualized that the intestinal microbiota species differed significantly between the two groups, while the species within the groups were similar. Figures 5B,C show that when only the presence or absence of microbial species in the community was considered, the differences between the two groups were more significant.

### 3.4. Diarrhea caused by cold and humid environmental stresses alters the structure of the intestinal contents microbiota

#### 3.4.1. Species composition at the phylum level

**Figure 6:**
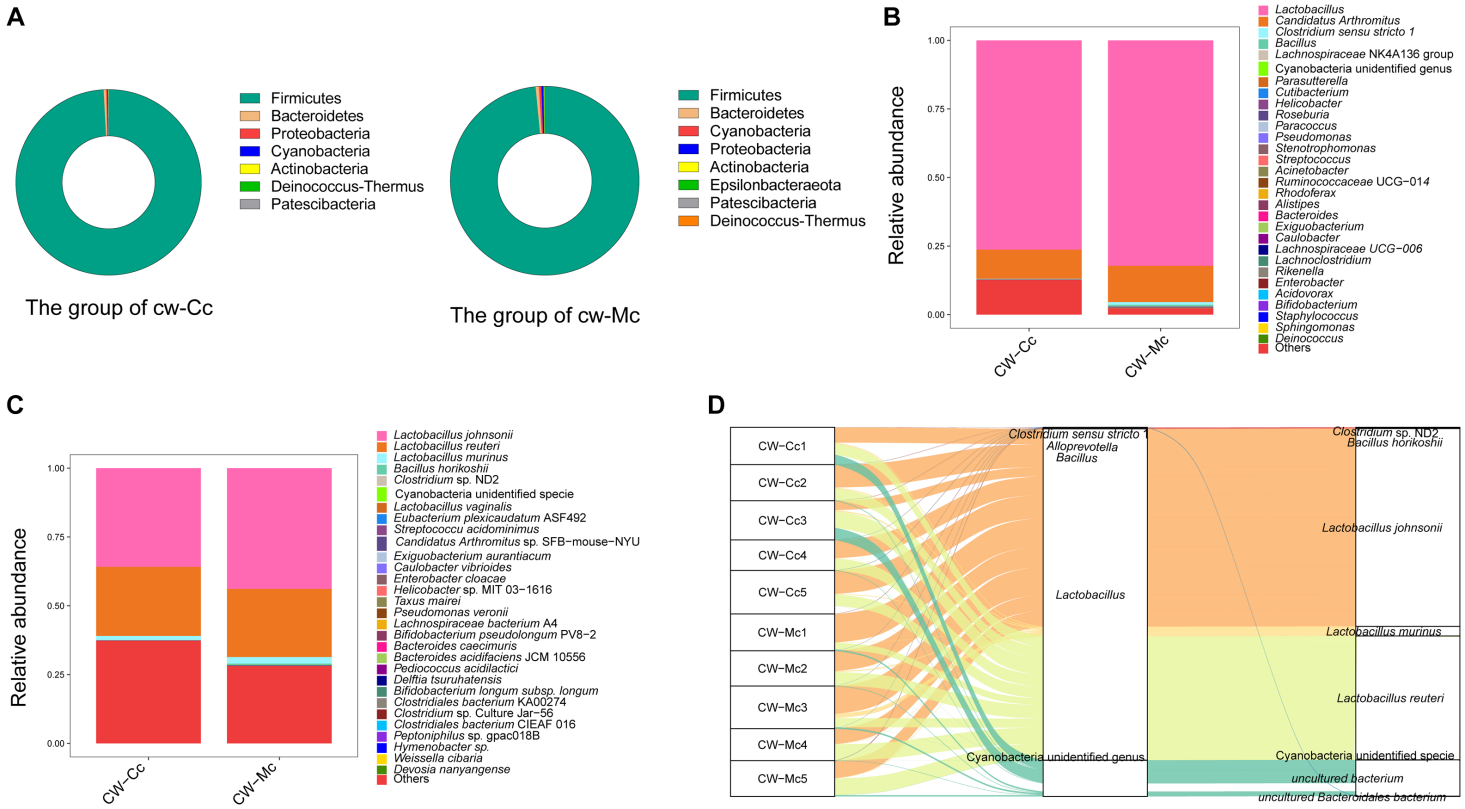
**(A)** The relative abundance of species at the phylum level. **(B)** The relative abundance of species at the genus level. **(C)** The relative abundance of species at the species level (“Others” indicates the sum of the relative abundance of all species other than these 30 species in the figure). **(D)** A heat map of the relative abundance of the top 30 species. The horizontal coordinate is the name of each sample, and the vertical coordinate is the name of the species; the closer to red, the higher the proportion of relative abundance of that species in the sample, and the closer to blue, the lower the proportion of relative abundance of the species in the sample.

Figure 6A illustrates the species composition and relative abundance at the phylum level. Firmicutes was overwhelmingly dominant in the CW-Cc and CW-Mc groups, accounting for 99.16% and 98.46%, respectively. In addition, Epsilonbacteraeota, a phylum specific to the CW-Mc group, was present in 80% of the samples.

#### 3.4.2. Species composition at the genus level

In order to identify the roles and contribution of all microbial taxa, we set thresholds as 0.01% for rare taxa and abundant taxa, and classified all OTUs into 2 exclusive categories based on their abundance. Our results demonstrated that the dominant genera in the CW-Cc group were *Lactobacillus* and *Candidatus Arthromitus*, while in the CW-Mc group were *Lactobacillus*, *Candidatus Arthromitus*, and *Clostridium sensu stricto* 1 (Figure 6B). As shown in Tables 1, 2, modeling increased the abundance of specific genera, including *Lactobacillus* (the CW-Cc group vs. the CW-Mc group: 76.3% vs. 82.2%), *Candidatus Arthromitus* (10.6% vs. 13.3%), *Clostridium sensu stricto* 1 (0.09% vs. 1%), and *Bacillus* (0.03% vs. 0.39%). In addition, more than 30 rare bacteria were stimulated to increase or appeared for the first time, such as *Helicobacter*, *Roseburia*, *Pseudomonas* etc.

**Table 1:** Relative abundance of abundant bacteria (genus level) (%).

Genus name	Normal group	Model group	Up or down
*Lactobacillus*	76.31473661	82.20493443	↑
*Candidatus arthromitus*	10.60680151	13.22516115	↑
*Clostridium sensu stricto* 1	0.088908646	0.995776839	↑
*Bacillus*	0.026672594	0.386752612	↑
*Lachnospiraceae* NK4A136 group	0.022227162	0.084463214	↑
Cyanobacteria unidentified genus	0.022227162	0.080017782	↑
*Stenotrophomonas*	0.008890865	0.048899756	↑
*Acinetobacter*	0.008890865	0.044454323	↑
*Paracoccus*	0.022227162	0.040008891	↑
*Streptococcus*	0.031118026	0.026672594	↓
*Cutibacterium*	0.057790620	0.022227162	↓

**Table 2:** Relative abundance of rare bacteria (genus level) (%).

Genus name	Normal group	Model group	Up or down
*Helicobacter*	0	0.066681485	↑
*Roseburia*	0	0.066681485	↑
*Pseudomonas*	0	0.05779062	↑
*Ruminococcaceae* UCG-014	0	0.040008891	↑
*Bacteroides*	0	0.031118026	↑
*Alistipes*	0	0.031118026	↑
*Exiguobacterium*	0	0.026672594	↑
*Caulobacter*	0	0.026672594	↑
*Lachnospiraceae* UCG-006	0	0.026672594	↑
*Rikenella*	0	0.026672594	↑
*Bifidobacterium*	0	0.022227162	↑
*Taxus mairei*	0	0.017781729	↑
*Brevundimonas*	0	0.017781729	↑
*Flavobacterium*	0	0.017781729	↑
*Nocardioides*	0	0.013336297	↑
*Lachnospiraceae* XPB1014 group	0	0.013336297	↑
*Candidatus Stoquefichus*	0	0.013336297	↑
*Candidatus Saccharimonas*	0	0.013336297	↑
*Roseomonas*	0	0.013336297	↑
*Oscillibacter*	0	0.008890865	↑
*Rheinheimera*	0	0.008890865	↑
*Agromyces*	0	0.008890865	↑
*Alloprevotella*	0	0.008890865	↑
*Clostridium sensu stricto 10*	0	0.008890865	↑
*Peptoniphilus*	0	0.008890865	↑
*Lachnospiraceae* UCG-001	0	0.008890865	↑
*Butyricicoccus*	0	0.008890865	↑
*Fastidiosipila*	0	0.008890865	↑
*Mesorhizobium*	0	0.008890865	↑
*Hymenobacter*	0.013336297	0	↓
*Agrococcus*	0.004445432	0	↓
*Hymenobacter*	0.013336297	0	↓
*Acetitomaculum*	0.004445432	0	↓
*Marvinbryantia*	0.004445432	0	↓
*Methylobacterium*	0.004445432	0	↓

#### 3.4.3. Species composition at the species level

Figure 6C and Table 3 illustrate that modeling increased the relative abundance of *Lactobacillus johnsonii* (the CW-Cc group vs. the CW-Mc group: 35.9% vs. 43.9%), *Lactobacillus murinus* (1.5% vs. 2.4%), Cyanobacteria unidentified specie (0.02% vs. 0.08%), and *Bacillus horikoshii* (0.02% vs. 0.37%), but decreased the relative abundance of *Lactobacillus reuteri* (25.1% vs. 24.8%) and *Clostridium* sp. ND2 (0.09% vs. 0.02%). As demonstrated in Table 4, the abundance of specific rare species disappeared after molding, such as of *Lachnospiraceae bacterium* A4, *Hymenobacter* sp., *Bacillus firmus*, *Firmicutes bacterium* CAG 194 44 15, *Clostridium* sp. Culture-54, *Methylobacterium radiotolerans*, and *Devosia nanyangense*, etc. However, more species were stimulated to grow after molding, including *Bifidobacterium longum* subsp. *longum*, *Taxus mairei*, *Deinococcus geothermalis* DSM 11300, *Helicobacter* sp. MIT 03–1,616, *Weissella cibaria*, *Clostridiales bacterium* CIEAF 016 etc.

**Table 3:** Relative abundance of abundant bacteria (species level) (%).

Species name	Normal group	Model group	Up or down
*Lactobacillus johnsonii*	35.86130251	43.88530785	↑
*Lactobacillus reuteri*	25.11224717	24.77439431	↓
*Lactobacillus murinus*	1.498110691	2.387197155	↑
*Clostridium* sp. ND2	0.088908646	0.017781729	↓
*Streptococcus acidominimus*	0.031118026	0.026672594	↓
Cyanobacteria unidentified specie	0.022227162	0.080017782	↑
*Lactobacillus vaginalis*	0.022227162	0.035563459	↑
*Bacillus horikoshii*	0.022227162	0.373416315	↑
*Candidatus Arthromitus* sp. SFB-mouse-NYU	0.013336297	0.017781729	↑

**Table 4:** Relative abundance of rare bacteria (species level) (%).

Species name	Normal group	Model group	Up or down
*Lachnospiraceae bacterium* A4	0.013336297	0	↓
*Hymenobacter* sp.	0.008890865	0	↓
*Bacillus firmus*	0.004445432	0	↓
*Firmicutes bacterium* CAG 194 44 15	0.004445432	0	↓
*Clostridium* sp. Culture-54	0.004445432	0	↓
*Methylobacterium radiotolerans*	0.004445432	0	↓
*Devosia nanyangense*	0.004445432	0	↓
*Bradyrhizobium japonicum*	0.004445432	0	↓
*Sphingomonas parapaucimobilis* NBRC 15100	0.004445432	0	↓
*Lachnoclostridium*	0.017781729	0.008890865	↓
*Enterobacter cloacae*	0.004445432	0.022227162	↑
*Bifidobacterium longum* subsp. *longum*	0	0.008890865	↑
*Bifidobacterium pseudolongum* PV8-2	0	0.013336297	↑
*Bacteroides acidifaciens*	0	0.004445432	↑
*Bacteroides acidifaciens* JCM 10556	0	0.013336297	↑
*Bacteroides caecimuris*	0	0.013336297	↑
*Elizabethkingia anophelis* PW2809	0	0.004445432	↑
*Taxus mairei*	0	0.017781729	↑
*Deinococcus geothermalis* DSM 11300	0	0.004445432	↑
*Deinococcus* sp.	0	0.004445432	↑
*Helicobacter* sp. MIT 03–1,616	0	0.017781729	↑
*Exiguobacterium aurantiacum*	0	0.026672594	↑
*Weissella cibaria*	0	0.004445432	↑
*Peptoniphilus* sp. gpac018B	0	0.008890865	↑
*Lachnospiraceae bacterium* COE1	0	0.004445432	↑
*Eubacterium plexicaudatum* ASF492	0	0.05779062	↑
*Clostridiales bacterium* CIEAF 016	0	0.008890865	↑
*Clostridium* sp. *Culture* Jar-56	0	0.008890865	↑
*Clostridiales bacterium* KA00274	0	0.008890865	↑

As shown in Figure 6D, the core species in both groups were *Lactobacillus johnsonii*, *Lactobacillus reuteri*, and *Lactobacillus murinus*, modeling did not significantly alter the abundance of the core species. Whereas, our analysis demonstrated that diarrhea mainly altered the composition structure and abundance of rare intestinal bacteria in mice, such as *Clostridium* sp. ND2 and *Bacillus horikoshii* etc.

### 3.5. Differential species analysis and network analysis of intestinal contents microbiota of diarrhea mice

**Figure 7:**
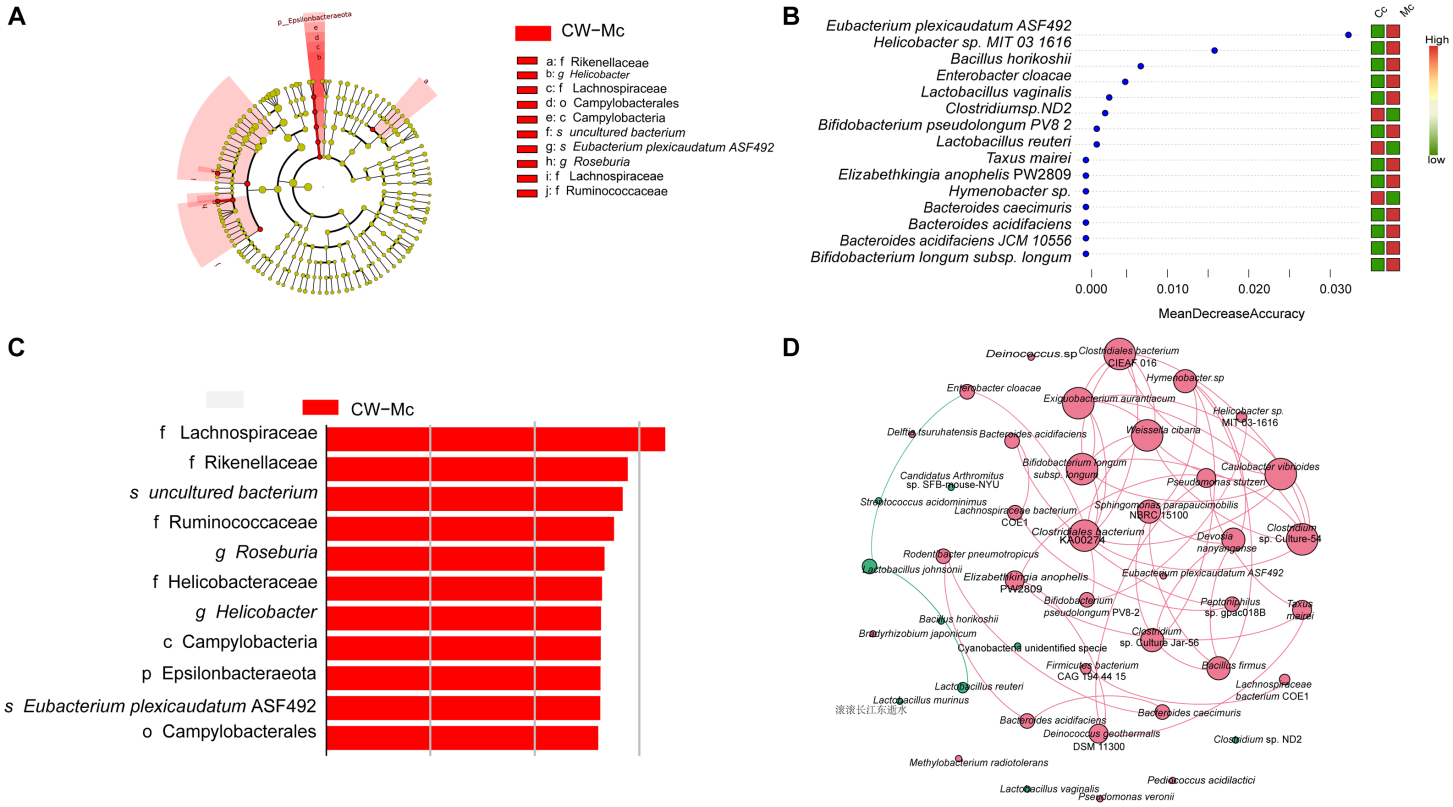
**(A)** Evolutionary branching diagram based on LDA Effect Size analysis showing the differential species in the model group. The colors indicate the different groupings, the red nodes in the branches indicate microbial taxa that play an important role in the red group, and the names of the species indicated by letters in the figure are shown in the legend. **(B)** The LDA Score greater than a set value (set to 2 by default). **(C)** Random forest variable importance plot. The abscissa is the mean decrease accuracy. The color red indicates that the species abundance grew in this group, whereas the color green shows that the species abundance dropped in this group. The variables are presented from descending importance. **(D)** Network analysis reveals the co-occurrence patterns of microbial taxa at the species level. Red nodes represent rare species and green nodes represent abundant species. The size of the nodes is proportional to the weights. Edges represent correlations between two species, red lines represent positive correlations, green lines represent negative correlations, the thickness of the lines is proportional to the correlation, and connections represent strong (*r* > 0.8) and significant (*p* < 0.05) correlations.

To further find potential biomarkers for diarrhea caused by cold and humid environmental stresses in mice, we conducted LEfSe analysis. As shown in Figures 7A,C, the differential species in the CW-Mc group were Campylobateria, Campylobaterales, Lachnospiraceae, Ruminococcaceae, Rikenellaceae, Helicobacteraceae, *Helicobacter*, *Roseburia*, and *Eubacterium plexicaudatum* ASF492. Random forest variable importance analysis can help predict which bacterial groups are critical in the CW-Mc group and may serve as distinctive markers for distinguishing the CW-Mc group from the CW-Cc group. Figure 7B shows that the bacteria that can best distinguish the two groups are *Eubacterium plexicaudatum* ASF492 and *Helicobacter* sp. MIT 031616, with MDA of 0.0322 and 0.0158, respectively. As can be seen from Figure 7D, network analysis reveals the co-occurrence patterns of microbial taxa at the species level. Our results showed that the interactions between abundant bacteria were less active than those between rare bacteria, there were essentially no interactions between abundant and rare bacteria. Besides, negative correlations were observed among abundant bacteria, such as *Lactobacillus johnsonii* and *Lactobacillus reuteri*, while positive correlations were observed among rare bacteria.

### 3.6. Bacterial function prediction analysis of intestinal contents microbiota of diarrhea mice

**Figure 8:**
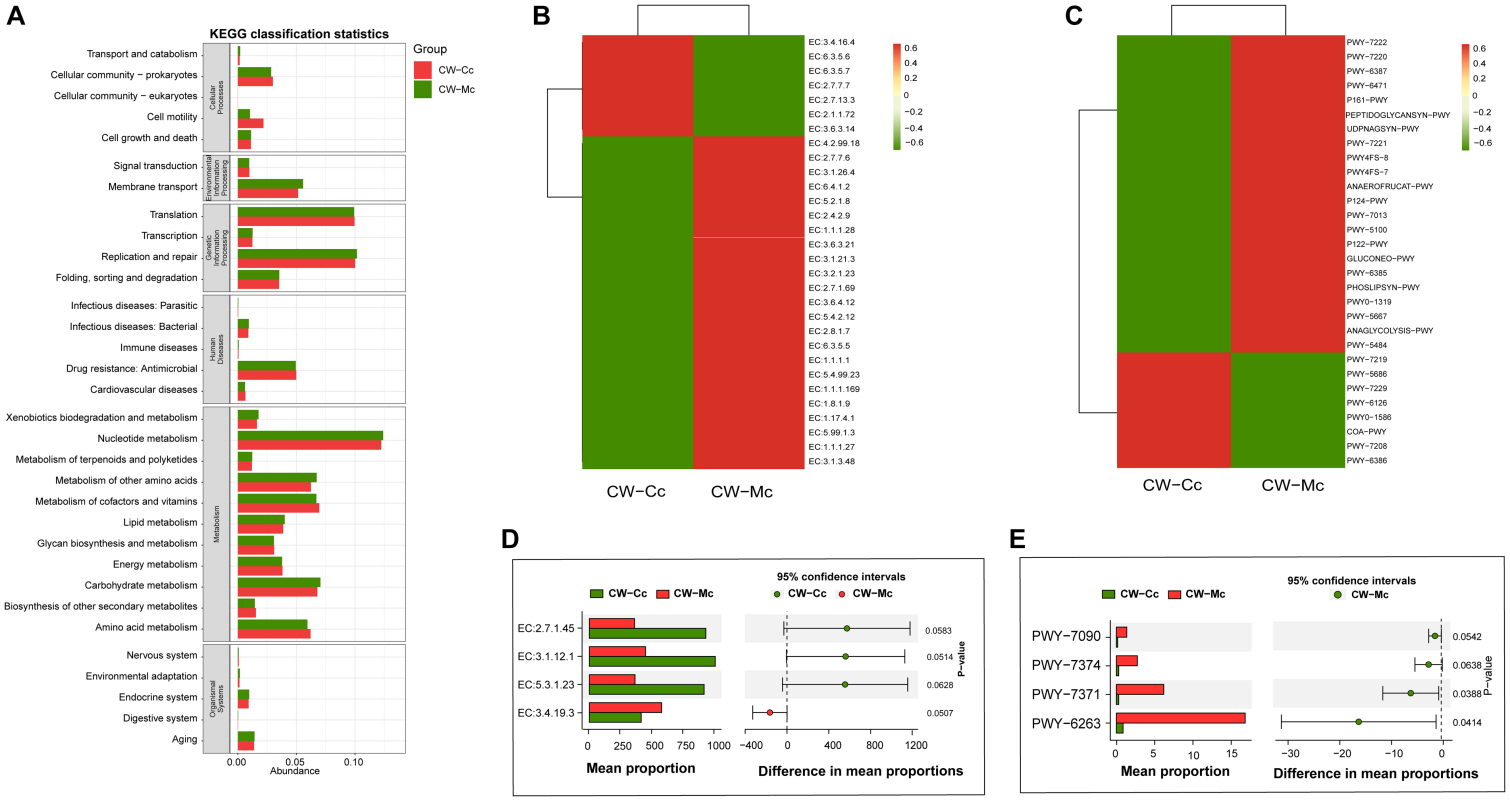
**(A)** The predicted KEGG secondary function abundance map. The abscissa is the relative abundance, the ordinate is the KEGG secondary functional classification, and the title of the gray area represents the name of the primary taxonomy corresponding to the secondary classification. **(B)** The relative abundance heat map (top 30) of the predicted enzymes. The abscissa is the grouping information, and the ordinate is the function information. The color corresponding to the middle heat map represents the *Z*-value obtained after normalization of the relative abundance of the function. The closer the color is to red, the higher the abundance. **(C)** The heat map (top 30) of the relative abundance of the pathway predicted based on the Metacy database. **(D)** The Stamp analysis (*T*-test) based on the relative abundance table of the enzyme. **(E)** The Stamp analysis (*T*-test) based on the pathway relative abundance table. The CW-Cc group is represented in red, and the CW-Mc group is represented in green.

As shown in Figure 8A, modeling stimulated secondary functions such as cell motility, amino acid metabolism, metabolism of cofactors and vitamins, and reduced the function of membrane transport. Furthermore, modeling mainly affected the abundance of oxidoreductases, hydrolases and transferases (Figures 8B,D). In the CW-Mc group, the synthesis of 2-dehydro-3-deoxygluconokinase (EC:2.7.1.45), 5′–3′ exodeoxyribonuclease (nucleoside 3′-phosphate-forming) (EC:3.1.12.1), S-methyl-5-thioribose-1-phosphate isomerase (EC:5.3.1.23) were inhibited, while the synthesis of pyroglutamyl-peptidase I (EC:3.4.19.3) was promoted. Finally, modeling made bacterial metabolic pathways more active. PWY-6263 and PWY-7371 were the differential metabolic pathways of the two groups (Figures 8C,E), which are related to the synthesis of Menaquinone 8, and modeling significantly promoted the synthesis of Menaquinone 8.

## 4. Discussion and conclusion

### 4.1. Diarrhea caused by cold and humid environmental stresses affects intestinal digestive enzyme activity and microbial activity

Digestive enzymes play a critical role in breaking down complex foods into simpler molecules like amino acids, glucose, and fatty acids. Once processed, these molecules are absorbed into the bloodstream, supplying the body with the energy and essential building blocks it requires for tissue regeneration. These enzymes predominantly reside in the host’s stomach, small intestine, and pancreas. Besides, certain digestive enzymes are either influenced by or are exclusively secreted by intestinal microorganisms. Amylase is mainly secreted by intestinal mucosa, sucrase and lactase can be either autonomously secreted or be products of intestinal microorganisms (Qiao et al., 2022). Microbial activity is a good indicator of microbial activity in the intestine (Tang et al., 2020). Our results showed a significant increase in lactase activity, sucrase activity and microbial activity in the intestine after the intervention of cold and humid environment, suggesting that the organism accelerates intestinal digestive capacity to store more energy to resist cold and humid environment stress, which also caused a response in the intestinal microbiota.

Lactase, a disaccharidase, has a specific role in hydrolyzing lactose within the small intestine. This enzyme can be located on the mucosal surface of the small intestinal mucosa’s brush border and to some extent in the intestinal contents (Zhou et al., 2023). Research has established that the Firmicutes phylum is the primary lactase-producing bacteria group (Wang et al., 2019). Additionally, entities like *Akkermansia muciniphila*, *Lactobacillus*, and the commensal *Bacteroides thetaiotaomicron* have been verified to produce β-galactosidase (Gil-Cruz et al., 2019; Kostopoulos et al., 2020). Further studies indicate that *Bacillus horikoshii* 20a, a unique Bacillus strain, possesses the capability to thrive in oligotrophic environments. This is achieved by efficiently storing and harnessing resources through the mechanism of *cyanobactin* metabolism (Zarza et al., 2017). In our experiment, the noted increase in *Bacillus* abundance in the mouse intestinal tract post-exposure to a cold and humid environment might be attributable to various factors. These include sudden spikes in food consumption, a swift elevation in enzyme activity, and a lack of optimal nutrient distribution within the intestines.

### 4.2. Diarrhea caused by cold and humid environmental stresses affects the diversity and species composition of the intestinal contents microbiota of mice

Epsilonbacteraeota, a distinct bacterial phylum found exclusively in the model group, originally known as Epsilonproteobacteria, was reclassified to better differentiate it from the traditional Gram-positive phylum proteobacteria (Waite et al., 2018). Capable of thriving under extreme conditions, such as high temperatures, pressures, and salinities, many Epsilonbacteraeota members can perform sulfide oxidation and other redox reactions, leveraging inorganic materials for energy. While some members form symbiotic relationships with animals and hold vital roles in ecosystems, others, like *Helicobacter pylori*, are known pathogens. In our research, extreme cold and humid conditions can alter gut nutrition and the symbiotic bacterial structure, potentially spurring Epsilonbacteraeota growth and increasing intestinal infection risks, potentially contributing to diarrhea in such environments. Besides, our results showed that the composition of the species after molding had two main characteristics. On the one hand, the cold and humid environmental stresses slightly changed the structure and composition of abundant bacteria in the intestine. At the genus and species level, we observed an increased relative abundance of *Lactobacillus*, *Candidatus Arthromitus*, *Clostridium sensu stricto* 1, *Lactobacillus johnsonii*, *Lactobacillus murinus*, Cyanobacteria unidentified specie, and *Bacillus horikoshii* within the intestinal tract. Notably, *Lactobacillus* and *Candidatus Arthromitus* are recognized for their probiotic functions, contributing to a healthy gut environment, while the majority of intestinal *Clostridium* species are involved in the metabolism of carbohydrates. The study indicates that *Bacillus horikoshii* may effectively store and utilize resources in oligotrophic environments through the *cyanobactin* metabolism mechanism (Zarza et al., 2017). This cooperative mechanism is likely responsible for the observed increase in the abundance of *Bacillus horikoshii* and Cyanobacteria unidentified specie in cold and humid environmental stresses in our study. Our study indicates a surge in intestinal enzyme activity under cold and humid stress. Consequently, this dynamic fosters a symbiotic rapport between the host and its intestinal microbiota, emphasizing the mutualistic benefits conferred to both entities. On the other hand, many rare bacteria grew significantly in the intestine, most of which are pathogenic or adaptable to extreme environments. *Helicobacter*, *Roseburia*. and *Pseudomonas* were unique genus in the model group, *Bifidobacterium longum* subsp. *longum*, *Helicobacter* sp. MIT 03–1616 and *Bacteroides acidifaciens* JCM 10556 were unique species in the model group. *Helicobacter* is an acid-tolerant bacteria that attacks the mucous membranes of the intestine, *Bacteroides acidifaciens* can increase fat oxidation in adipose tissue through the peroxisome prolife rator-activated receptor-α (PPARα) (Yang et al., 2017). *Pseudomonasa* is a typical spoilage bacterium that can tolerate extreme physical conditions, such as high humidity and low temperatures environment (Dogan and Boor, 2003). Studies have shown that *Pseudomonasa* can promote the synthesis of O antigen, the main component of lipopolysaccharide (Dasgupta et al., 1994; Burrows et al., 1996), and our function prediction results show that PWY-7090, which is related to O antigen synthesis, is specifically increased in the model group, which is consistent with the increase in the abundance of *Pseudomonasa*, indicating that an increase in *Pseudomonasa* may negatively affect host physiological functions.

Although the microbial communities in the environment are highly diverse, the dominant species (i.e., high abundance species) are few, most species are rare bacteria (relative abundance less than 0.1% or even 0.01%) (Yang et al., 2022). Study show that psychrophilic organisms have evolved different strategies to cope with the extreme environmental stresses. Low-temperature extremophiles modify the lipid composition of cell membranes to maintain mobility and enter a dormant state to limit metabolic activity (Shu and Huang, 2022), and cryophilic proteins can increase the catalytic efficiency after active site modifications that maintain a low but sufficient level of metabolism for cytokinesis (Cavicchioli et al., 2002).

### 4.3. Diarrhea caused by cold and humid environmental stresses changes the interaction patterns among intestinal bacteria

More interestingly, our study revealed a phenomenon that rare and abundant bacteria chose different interaction patterns under the cold and humid environmental stresses. Network analysis shows the interactions within the abundant species may be mainly competitive or inhibitory, such as *Lactobacillus johnsonii* and *Lactobacillus reuteri*. In contrast, rare species reinforce each other’s cooperative relationships to cope with nutrient deficiencies to resist cold and humid environments. Studies have shown that most abundant microbial and rare microbial have significant differences in competitive strategies and environmental adaptation, but there is no clear understanding of how biotic and abiotic factors influence the competitive strategies in which different bacterial communities interact with each other. Dai et al. (2022) has found that high-growth bacteria dominate, slow-growth bacteria are excluded from competition in eutrophic environments, but microorganisms tend to cooperate in reducing resource shortages and maintaining life processes in oligotrophic environments. Wu et al. (2020) has found that the growth of a large number of rare bacteria complicates the collaboration between intestinal bacteria and improves the use of nutrients. Cold and humid environmental stresses accelerate intestinal mucous membrane energy consumption, diarrhea reduces the residence time of nutrients in the small intestine, and lack of nutrients in the intestine. In this situation, rare bacteria that grow slowly increase interactions to improve the efficiency of the utilization of nutrients in the intestine, together promoting growth, but abundant bacteria choose alternative nutritional strategies: negative relationships between interactions. We speculate that, diarrhea caused by cold and humid environmental stresses leads indirectly to an oligotrophic environments in the intestine, with rare bacteria choosing to improve their interaction and help each other in response to the new environment, while abundant bacteria choosing to compete and inhibit with each other, such as *Lactobacillus johnsonii* and *Lactobacillus reuteri*.

In summary, our study find that cold and humid environmental stress affects the nutritional environment in the intestine, mainly altering the abundance and species composition of rare bacteria in the intestine, and rare bacteria such as *Clostridium sensu stricto* 1 and *Helicobacter* have the potential to be model intestine biomarkers. Modeling changed the survival strategy and cooperation between abundant and rare bacteria, and the relationship between *Lactobacillus johnsonii* and *Lactobacillus reuteri* (abundant bacteria) was competitive or inhibitory, while rare bacteria strengthened mutual cooperation. Our study provides a basis for further understanding the interaction of intestinal microbiota and accurate diagnosis of diarrhea with cold-dampness trapped spleen syndrome in TCM.

However, our research is somewhat limited, we used content samples from two groups of mice instead of a single mouse sample to compare digestive enzyme activities, which limited the correlation analysis between digestive enzymes and intestinal bacteria. In addition, we did not find a corresponding relationship between digestive enzymes, bacterial abundance, and function prediction results, indicating that the intestinal environment of mice under cold and damp stress conditions is very complex, and we should consider the influence of single factors on the intestinal content microbiota.

## Data availability statement

The datasets presented in this study can be found in online repositories. The names of the repository/repositories and accession number(s) can be found at: https://www.ncbi.nlm.nih.gov/, PRJNA797112, https://www.ncbi.nlm.nih.gov/, PRJNA797198.

## Ethics statement

The animal study was approved by the Experimental Animal Ethics Committee of Hunan University of Chinese Medicine. The study was conducted in accordance with the local legislation and institutional requirements.

## Author contributions

YW: Data curation, Methodology, Visualization, Writing – original draft. ND: Data curation, Project administration, Supervision, Writing – review & editing. JL: Formal analysis, Visualization, Writing – original draft. PJ: Conceptualization, Funding acquisition, Supervision, Writing – review & editing. ZT: Conceptualization, Supervision, Writing – review & editing, Funding acquisition.
